# Risk Factors for COVID-19 Cluster Infection in Hospitalized Patients

**DOI:** 10.7759/cureus.57957

**Published:** 2024-04-10

**Authors:** Yoritake Sakoda, Takanori Matsumoto, Masaki Yamaguchi, Asuka Kudo, Kumiko Nakano, Yasuki Maeno

**Affiliations:** 1 Infectious Diseases, St. Mary’s Hospital, Kurume, JPN; 2 Pharmacy, St. Mary’s Hospital, Kurume, JPN; 3 Safety and Infection Control, St. Mary’s Hospital, Kurume, JPN; 4 Medical Quality Management Center, St. Mary’s Hospital, Kurume, JPN

**Keywords:** infection control, infection prevention, sars-cov-2, cluster infection, coronavirus disease 2019 (covid-19)

## Abstract

Introduction

In Japan, in the seventh wave of coronavirus disease 2019 (COVID-19) from July 2022 to September 2022, followed by the eighth wave of COVID-19 from November 2022 to January 2023, nosocomial clusters became more frequent in many healthcare facilities. If a cluster occurs in a hospital, the restrictions on general healthcare and the impact on hospital management, as well as the impact on community healthcare, are enormous. We analyzed the risk factors for COVID-19 cluster infection in hospitalized patients.

Methods

We retrospectively collected cases of COVID-19 infection among hospitalized patients in the seventh and eighth waves. The occurrence of a COVID-19 patient in a hospitalized patient was defined as one event.

Results

A total of 40 events were observed in the seventh and eighth waves. There were 17 events that developed into clusters. The following factors showed a significant association with cluster infection in a univariate analysis: “seventh wave,” “originated from healthcare worker,” and “initial examination according to contact list.” The multivariate analysis revealed that “originated from healthcare worker” was independently associated with cluster infection.

Conclusion

Preventing the development of COVID-19 clusters is very important for nosocomial infection control. Our study suggests that COVID-19 infection in a healthcare worker is a risk factor for the development of a cluster. When healthcare workers are infected with severe acute respiratory syndrome coronavirus 2 (SARS-CoV-2), it is often due to household transmission. Measures against household transmissions are important to prevent infection among healthcare workers.

## Introduction

Coronavirus disease 2019 (COVID-19), which started at the end of 2019, has repeatedly mutated and become more infectious [1]. In Japan, in the seventh wave from July 2022 to September 2022, followed by the eighth wave from November 2022 to January 2023, nosocomial clusters became more frequent in many healthcare facilities [2]. The main variant in the seventh wave and the eighth wave was Omicron BA.5. If a cluster occurs in a hospital, many healthcare workers as well as patients will be infected and the workforce will be short-staffed. This may result in ward closures, with significant restrictions on general healthcare, an impact on hospital management, and immeasurable consequences for community healthcare.

Our hospital is the core hospital of the Kurume medical region, which has a population of approximately 450,000. The hospital is a regional medical support hospital with 41 departments, 1,097 beds, and 2,400 staff, and plays a particularly important role in emergency medicine. More than 30,000 emergency patients are treated annually, among which approximately 10,000 are ambulance admissions. The cessation of a hospital’s medical functions due to the occurrence of a COVID-19 cluster would have a very significant impact on local healthcare.

Japan intended to change the classification of COVID-19 under the Infectious Diseases Act to category 5, equivalent to seasonal influenza, on May 8, 2023, thereby easing social restrictions [3]. However, the easing of restrictions does not weaken the infectivity of the virus, which means that nosocomial infection control measures will be more difficult than before. Preventing the development of COVID-19 clusters is very important for nosocomial infection control. In infection control measures during COVID-19 clusters, studies have examined the risk of infection among healthcare workers [4], but research investigating the factors contributing to cluster outbreaks is lacking. Based on our experience from the seventh and eighth waves, we analyzed the risk factors for COVID-19 cluster infection in hospitalized patients.

## Materials and methods

We retrospectively collected data on patients admitted to St. Mary's Hospital, Kurume, Japan, who contracted COVID-19 during their hospitalization, and the risk factors associated with the development of clusters were analyzed. Data of the seventh wave (July 1 to September 30, 2022) and the eighth wave (November 1, 2022 to January 31, 2023) were collected from the COVID-19 patient trends database of the Infection Control Unit of St. Mary's Hospital. The occurrence of a COVID-19 infection in a hospitalized patient was defined as one event. The diagnosis of COVID-19 was based on a positive antigen quantification test or polymerase chain reaction (PCR) test for severe acute respiratory syndrome coronavirus 2 (SARS-CoV-2). A cluster was defined as more than five cases of COVID-19 infection among hospitalized patients or healthcare workers during one event. The start of one event was defined as the time at which there was a known case of COVID-19 infection in a hospitalized patient, and the end of one event was defined as when no new infections appeared for one week from the last hospitalized patient or healthcare worker. Healthcare workers included doctors, nurses, care workers, and physical therapists. The initial epidemiological examination at the time of the event was conducted according to a contact list or an extended examination. In an epidemiological examination according to a contact list, in the event of COVID-19, a list is drawn up specifying the degree of contact going back to the previous two days, and a decision is made as to whether an epidemiological examination should be carried out according to the degree of contact described on the list. Extended examination is a method whereby, in the event of COVID-19, all healthcare workers and patients in the ward in which COVID-19 infection occurred are investigated, regardless of the degree of contact. Statistical analyses were performed using the JMP® software program (SAS Institute Inc., Cary, NC, USA). Bivariate comparisons between groups were performed using independent t-tests and chi-square tests, as appropriate. Logistic regression was used to identify independent predictors of risk factors. This present study received approval from the Ethical Review Board of our hospital.

## Results

As shown in Table 1, a total of 40 events were observed in the seventh and eighth waves. The recognized events included 11 events in the seventh wave and 29 events in the eighth wave. There were 17 events that developed into clusters and 23 that did not. Events that developed into clusters were more common in the seventh wave than in the eighth wave (Table 2). Healthcare worker-originated events were more common in the clusters. The type of ward (medical or surgical ward) in which cluster infection occurred was compared between the cluster and non-cluster groups but did not differ to a statistically significant extent. In the cluster group, the initial examination often followed the contact list, while in the non-cluster group, extended examinations were carried out more frequently.

**Table 1:** Demographics and characteristics of each event. COVID-19, coronavirus disease 2019; extended, all healthcare workers and patients in the ward in which COVID-19 infection occurred are investigated, regardless of the degree of contact

No.	Wave	Origin	Ward	Infected patients	Infected healthcare workers	Days until the event ends	Initial examination
1	Seventh	Patient	General Surgery	15	18	26	According to contact list
2	Seventh	Healthcare worker	Orthopedic Surgery	19	13	27	According to contact list
3	Seventh	Healthcare worker	Plastic Surgery	11	14	20	According to contact list
4	Seventh	Unknown	Gastroenterology	7	1	17	According to contact list
5	Seventh	Patient	Nephrology/Endocrinology	11	5	23	According to contact list
6	Seventh	Patient	Urology/Otorhinolaryngology	14	7	19	According to contact list
7	Seventh	Healthcare worker	Neurology	6	6	15	According to contact list
8	Seventh	Healthcare worker	Neurological Surgery	22	13	13	According to contact list
9	Seventh	Unknown	High Care Unit	1	0	7	Extended
10	Seventh	Unknown	Cardiology	1	0	7	According to contact list
11	Seventh	Unknown	Obstetrics	1	0	7	According to contact list
12	Eighth	Healthcare worker	Neurological Surgery	8	2	21	Extended
13	Eighth	Healthcare worker	Cardiology	1	1	7	Extended
14	Eighth	Patient	Ophthalmology	1	0	7	Extended
15	Eighth	Patient	Gastroenterology	1	0	7	Extended
16	Eighth	Unknown	General Surgery	1	0	7	Extended
17	Eighth	Healthcare worker	General Surgery	2	3	10	Extended
18	Eighth	Unknown	General Surgery	8	0	12	Extended
19	Eighth	Unknown	Gastroenterology	3	0	7	Extended
20	Eighth	Patient	Pediatrics	2	2	8	Extended
21	Eighth	Healthcare worker	Urology/Otorhinolaryngology	1	2	8	According to contact list
22	Eighth	Healthcare worker	Nephrology/Endocrinology	3	2	11	According to contact list
23	Eighth	Healthcare worker	Neurological Surgery	5	1	15	According to contact list
24	Eighth	Healthcare worker	Cardiology	11	1	20	According to contact list
25	Eighth	Healthcare worker	Psychiatry	5	5	12	Extended
26	Eighth	Healthcare worker	Orthopedic Surgery	1	1	7	Extended
27	Eighth	Patient	Hematology	1	0	7	According to contact list
28	Eighth	Healthcare worker	High Care Unit	1	1	7	According to contact list
29	Eighth	Unknown	General Surgery	3	0	12	Extended
30	Eighth	Healthcare worker	Intensive Care Unit	1	1	7	Extended
31	Eighth	Unknown	Urology/Otorhinolaryngology	2	4	10	According to contact list
32	Eighth	Unknown	Gastroenterology	2	1	13	Extended
33	Eighth	Unknown	Plastic Surgery	8	1	10	Extended
34	Eighth	Unknown	Gynecology	1	0	7	Extended
35	Eighth	Unknown	Psychiatry	1	0	7	Extended
36	Eighth	Unknown	Neurology	1	0	7	Extended
37	Eighth	Unknown	Hematology	1	0	7	Extended
38	Eighth	Unknown	Neurological Surgery	4	0	8	Extended
39	Eighth	Unknown	Orthopedic Surgery	1	0	7	Extended
40	Eighth	Unknown	High Care Unit	1	0	7	Extended

**Table 2:** Comparison of cluster group and non-cluster group. COVID-19, coronavirus disease 2019; cluster group, more than five cases of COVID-19 infection among hospitalized patients or healthcare workers during one event; non-cluster group, less than five cases of COVID-19 infection among hospitalized patients or healthcare workers during one event; number of infected cases, average number in one event ^a^χ^2^ test; ^b^t-test, ※p<0.05

Characteristics	Cluster group, n＝17	Non-cluster group, n＝23	p-Value
Wave of COVID-19			
Seventh	8 (47.0%)	3 (13.0%)	0.031^a※^
Eighth	9 (53.0%)	20 (87.0%)	0.031^a※^
Origin of infection			
Patient	3 (17.6%)	4 (17.4%)	1.000^a^
Healthcare worker	10 (58.8%)	5 (21.7%)	0.023^a※^
Unknown	4 (23.5%)	14 (60.9%)	0.027^a※^
Ward			
Medical ward	7 (41.2%)	10 (43.5%)	1.000^a^
Surgical ward	10 (58.8%)	13 (56.5%)	1.000^a^
Number of infected cases			
Patient	9.235±5.717	1.391±0.838	<0.001^b※^
Healthcare worker	5.647±5.522	0.3913±0.656	<0.001^b※^
Days until the event ends	16.529±5.636	7.608±1.587	<0.001^b※^
Initial examination			
According to contact list	12 (70.6%)	5 (21.7%)	0.003^a※^
Extended	5 (29.4%)	18 (78.3%)	0.003^a※^

A univariate analysis was performed to analyze the risk factors associated COVID-19 cluster infection in hospitalized patients. As shown in Table 3, the following factors showed a significant association with cluster infection: “seventh wave,” “originated from healthcare worker,” and “initial examination according to contact list.” A multivariate analysis was performed using these parameters, which revealed that “originated from healthcare worker” was independently associated with cluster infection (Table 4).

**Table 3:** Univariate analysis for risk factors associated with COVID-19 clusters. COVID-19, coronavirus disease 2019 ¶Calculated by logistic regression analysis; ※p<0.05

Variables	OR	95% CI	p-Value^¶^
Wave of COVID-19			
Seventh	5.93	1.27-27.7	0.024^※^
Eighth	0.17	0.04-0.79	0.024^※^
Origin of infection			
Patient	1.02	0.20-5.29	0.983
Healthcare worker	5.14	1.29-20.5	0.02^※^
Unknown	0.2	0.05-0.8	0.023^※^
Ward			
Medical ward	0.91	0.26-3.24	0.884
Surgical ward	1.1	0.31-3.91	0.884
Initial examination			
According to contact list	8.64	2.05-36.4	0.003^※^
Extended	0.116	0.03-0.49	0.003^※^

**Table 4:** Multivariate analysis for risk factors associated with COVID-19 clusters. COVID-19, coronavirus disease 2019 ¶Calculated by logistic regression analysis; ※p<0.05

Variables	OR	95% CI	p-Value^¶^
Seventh wave	3.9	0.46-33.4	0.215
Originated from healthcare worker	5.55	1.03-29.9	0.045^※^
Initial examination according to contact list	3.55	0.56-22.3	0.177

## Discussion

We analyzed the risk factors for COVID-19 cluster infection in hospitalized patients. The multivariate analysis revealed that “originated from healthcare worker” was independently associated with cluster infection. One healthcare worker may have contact with several patients. Contact with several patients or prolonged contact is a risk factor for COVID-19 transmission [5], and the presence of a healthcare worker with COVID-19 is considered to be a risk factor for cluster infection. Based on our research findings, it can be concluded that preventing healthcare worker infections is crucial to preventing nosocomial clusters of COVID-19.

When healthcare workers are infected with SARS-CoV-2, it is often due to household transmission [6,7]. Because COVID-19 is highly contagious, the risk of household transmission is high [8]. In our hospital survey, approximately 80% of healthcare workers who served as infection sources were found to have acquired the infection through household transmission. This indicates the importance of measures against household transmission in preventing nosocomial clusters of COVID-19. In our hospital, it is standard practice to isolate the infected family member at home when a healthcare worker's family member contracts COVID-19. Additionally, in such cases, healthcare workers are considered close contacts, and immediate antigen quantitative testing is conducted to determine whether they have been infected with SARS-CoV-2. We rigorously implement infection control measures from the early stages for healthcare workers. However, it is difficult to prevent household transmission in practice, as healthcare workers often already test positive at the time that a family member is found to have COVID-19. COVID-19 is contagious even when asymptomatic [9], which means that even if a rapid response is taken to detect a household infection at an early stage, the healthcare worker has already become a source of infection. In view of this, healthcare workers should always implement measures to minimize the risk of transmission to others, even if they are unaware of being infected with COVID-19 themselves. This generally involves strict adherence to universal masking and standard precautions [10]. The main route of transmission of COVID-19 is through droplet transmission. It is said that wearing a mask can reduce the spread of infection [11]. Furthermore, it has been reported that the risk of infection transmission can be further reduced by wearing masks with each other [12]. If the other person is not wearing a mask, eye protection is recommended [9]. Universal masking is always practiced by all healthcare workers in our hospital. However, healthcare workers were not thorough in ensuring that patients wore masks during consultations. Furthermore, the wearing of face shields during suctioning and meal assistance, when the patient is unmasked, was not thorough. We instruct healthcare workers to encourage patients to wear masks during patient care and to ensure that face shields are worn in all necessary situations. Although contact transmission is said to carry a lower risk of transmission than droplet transmission [13], contact transmission may well occur, depending on the extent of patient care. The overall hand hygiene compliance rate in our hospitals is around 60%, and we are making announcements to further increase hand hygiene compliance. We regularly instruct healthcare workers on the proper use of appropriate personal protective equipment based on the nature of patient care, and compliance has been relatively good. To prevent nosocomial clusters of COVID-19, we believe that it is crucial to rigorously implement universal masking and standard precautions even during non-outbreak periods.

When comparing the seventh and eighth waves, more clusters were found in the seventh wave. The initial epidemiological examination of the seventh wave also included a number of examinations according to contact list. Examinations according to contact list focused on close contact with COVID-19 patients for more than 15 minutes, but this did not prevent progression to clusters. Early detection, contact tracing, and quarantine are important in cluster control [14,15]. In the eighth wave, many extended examinations were carried out to detect infected individuals at an early stage. To prevent nosocomial clusters of COVID-19, it may be necessary to conduct epidemiological investigations across a wide range from the early onset of cases.

There are several limitations in this study. First, we were unable to obtain information on the patient's activities of daily living (ADL). Patients with low ADL come into frequent contact with healthcare workers and are thought to be at higher risk of infection. Second, this study did not consider patient severity. It is said that the period of virus shedding may be longer than usual depending on the patient's severity and immune status [16-18], and this is also considered to be a risk factor for infection. We do not have records of these patient factors in our database and could not evaluate them. In the future, we need to improve our data collection methods.

## Conclusions

In Japan's COVID-19 infection control, social mitigation is likely to increase the opportunities for household transmission among healthcare workers. Our study suggests that COVID-19 infection in a healthcare worker is a risk factor for the development of a cluster. Measures against household transmissions are important to prevent infection among healthcare workers. However, there are limitations in preventing household transmission. To prevent nosocomial clusters of COVID-19, it is crucial not only to prevent COVID-19 infections among healthcare workers but also to adhere to universal masking and standard precautions during non-outbreak periods, and to conduct extended epidemiological investigations from the early stages in the event of COVID-19 patient occurrence.
